# DIET@NET: Best Practice Guidelines for dietary assessment in health research

**DOI:** 10.1186/s12916-017-0962-x

**Published:** 2017-11-15

**Authors:** Janet E. Cade, Marisol Warthon-Medina, Salwa Albar, Nisreen A. Alwan, Andrew Ness, Mark Roe, Petra A. Wark, Katharine Greathead, Victoria J. Burley, Paul Finglas, Laura Johnson, Polly Page, Katharine Roberts, Toni Steer, Jozef Hooson, Darren C. Greenwood, Sian Robinson, on behalf of the DIET@NET consortium, Nisreen Alwan, Janet Cade, Paul Finglas, Tim Key, Barrie Margetts, Andrew Ness, Sian Robinson, Toni Steer, Polly Page, Petra Wark

**Affiliations:** 1 Nutritional Epidemiology Group, School of Food Science and Nutrition, University of Leeds, Leeds, LS2 9JT UK; 2 Department of Food Science and Nutrition, King Abdulaziz University, PO Box 42807, 21551 Jeddah, Saudi Arabia; 3 Academic Unit of Primary Care and Population Sciences, Faculty of Medicine, University of Southampton, Southampton General Hospital, Southampton, SO16 6YD UK; 4 NIHR Biomedical Research Unit in Nutrition, Diet and Lifestyle, University Hospitals Bristol NHS Foundation Trust and the University of Bristol, Bristol, BS8 1TH UK; 5 Quadram Institute Bioscience, Norwich, NR4 7UA UK; 6 Centre for Innovative Research Across the Life Course (CIRAL), Faculty of Health and Life Sciences, Coventry University, Coventry, CV1 5FB UK; 7 Global eHealth Unit, Department of Primary Care and Public Health, Imperial College London, London, SW7 2AZ UK; 8 Centre for Exercise, Nutrition and Health Sciences, School for Policy Studies, University of Bristol, Bristol, BS8 1TH UK; 9 MRC Elsie Widdowson Laboratory, Cambridge, CB1 9NL UK; 10 Public Health Section, School of Health and Related Research (ScHARR), University of Sheffield, Sheffield, S10 2TN UK; 11 Public Health England, London, SE1 8UG UK; 12 Faculty of Medicine and Health, Division of Biostatistics, University of Leeds, Leeds, LS2 9JT UK; 13 MRC Lifecourse Epidemiology Unit, University of Southampton, Southampton, SO16 6YD UK; 14 NIHR Southampton Biomedical Research Centre, University of Southampton & University Hospital Southampton NHS Foundation Trust, Southampton, SO16 6YD UK

**Keywords:** Dietary assessment methods, Guidelines, Nutritional epidemiology, Nutrition, Public health

## Abstract

**Background:**

Dietary assessment is complex, and strategies to select the most appropriate dietary assessment tool (DAT) in epidemiological research are needed. The DIETary Assessment Tool NETwork (DIET@NET) aimed to establish expert consensus on Best Practice Guidelines (BPGs) for dietary assessment using self-report.

**Methods:**

The BPGs were developed using the Delphi technique. Two Delphi rounds were conducted. A total of 131 experts were invited, and of these 65 accepted, with 48 completing Delphi round I and 51 completing Delphi round II. In all, a total of 57 experts from North America, Europe, Asia and Australia commented on the 47 suggested guidelines.

**Results:**

Forty-three guidelines were generated, grouped into the following four stages: Stage I. Define what is to be measured in terms of dietary intake (what? who? and when?); Stage II. Investigate different types of DATs; Stage III. Evaluate existing tools to select the most appropriate DAT by evaluating published validation studies; Stage IV. Think through the implementation of the chosen DAT and consider sources of potential biases.

**Conclusions:**

The Delphi technique consolidated expert views on best practice in assessing dietary intake. The BPGs provide a valuable guide for health researchers to choose the most appropriate dietary assessment method for their studies. These guidelines will be accessible through the Nutritools website, www.nutritools.org.

**Electronic supplementary material:**

The online version of this article (doi:10.1186/s12916-017-0962-x) contains supplementary material, which is available to authorized users.

## Background

Accurate assessment of dietary exposure is challenging [1] due to differences between populations and the amount and kind of food consumed, which varies day to day between and within study participants and over the life course. With more than 45,000 products on our supermarket shelves, people may not know exactly what they have eaten and how much they have consumed. The availability and accessibility of different foods may also influence dietary patterns [2].

Key challenges for self-reported dietary assessment tools (DATs) relate to measurement error and validation of methods, and it has been acknowledged that none of the dietary assessment methods available for measuring dietary intake are totally free of error [3, 4]. Some dietary assessment instruments will be more prone to error than others, and tools will have varying degrees of random and systematic errors. Researchers may choose a particular instrument for practical reasons, such as cost. However, if the error is not acknowledged, results may be misleading [5]. Thus, accurate approaches to assessment of dietary intake are needed [6]. Dietary intake can be assessed by subjective self-report such as food diaries, recalls or food frequency questionnaires (FFQs), with each different assessment approach having its own limitations, or by use of an alternative objective method (e.g. nutrient biomarkers [7]), which may have less error than self-reported estimated intakes [6, 8]. However, results based on biochemical values are limited to a few nutrients, and they cannot capture which foods and beverages were consumed.

Measurement error in dietary assessment can create spurious associations in epidemiology [9, 10]. Under-reporting has long been demonstrated in National Diet and Nutrition Surveys, with higher levels among less well educated and overweight or obese populations [11].

Systematic reviews of diet and health are affected by substantial heterogeneity, resulting in part from use of less than optimal measurement tools. For example, a review of studies reporting sodium intake linked to cardiovascular disease outcomes concluded that methodological issues accounted for the inconsistent findings [12]. The quality of various approaches to measure food and nutrient intakes varies along with their suitability in particular situations. Currently there is reliance on self-reporting, and the selection of a tool strongly depends upon the study design; thus, guidance for researchers is urgently needed. Therefore, development of strategies that support researchers to choose the most appropriate dietary assessment method will help to strengthen research in this field and the quality of findings underpinning diet and disease relationships.

The DIETary Assessment Tool NETwork (DIET@NET) partnership project aimed to establish Best Practice Guidelines (BPGs) to help non-expert researchers in dietary assessment select the most appropriate DAT. This paper summarises the process of developing the BPGs, as well as the guidelines themselves, with brief explanations of the statements and guidance for their use. The BPGs will be available interactively, with further detail, through the Nutritools website (www.nutritools.org). These guidelines should be used by researchers when planning studies involving self-reported dietary measurement.

## Methods

**Fig. 1:**
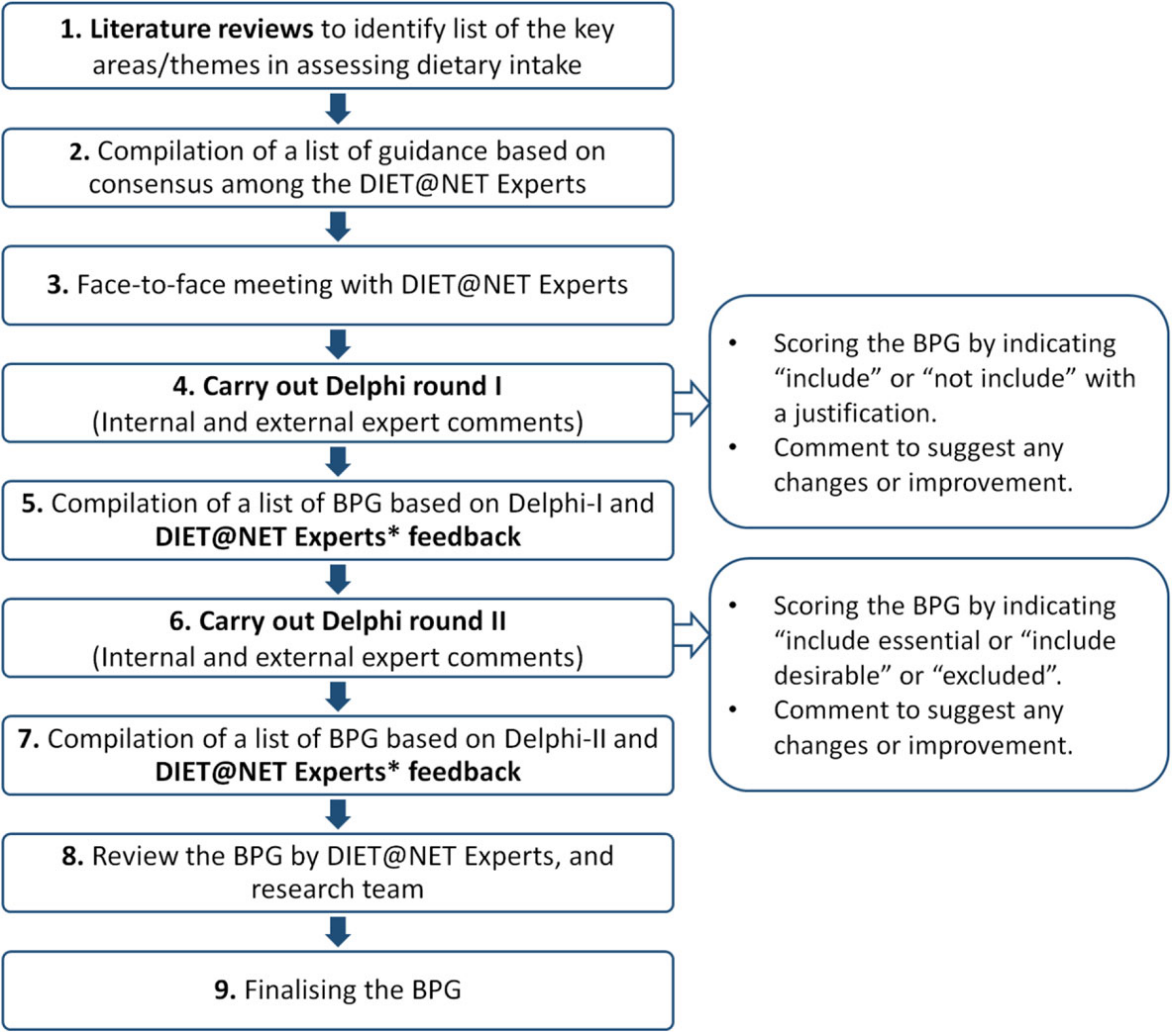
Steps for the development of the Best Practice Guidelines for dietary assessment

The BPGs for dietary assessment were developed using a modified Delphi technique. This approach uses a multistage, self-completed questionnaire with individual feedback from ‘experts’ to reach consensus [13]. Figure 1 summarises the BPG development process. We started development of the BPGs by compiling a preliminary list of guidelines developed from a search of academic literature including Web of Science and Ovid MEDLINE. This search was conducted, using a non-systematic approach, in 2015 by the DIET@NET consortium research team. The literature review included key considerations when evaluating the choice of the best approach to collect dietary data. An initial set of minimum requirements for quality standards was prepared based on the literature including population studied, dietary intake measurement, tool choice, nutrient information and aspects relating to analysis and validation of tools [14]. The focus of the guidelines was on practical use, and the development phase also included scenario testing with an example study design. This helped to revise the headings, wording and order of the guidelines. An expert group from the DIET@NET project partners revised and updated the preliminary guidelines in two stages: firstly via email exchanges, followed by a face-to-face meeting. The proposed BPGs were circulated to a wider group of experts by a self-completion structured questionnaire, in Delphi rounds. The focus was collection of dietary data using self-report and did not specify particular methodologies. However, we did ask for comments on strengths and weaknesses of the following methods: food diaries, 24-hour recalls (24HRs), FFQs, food checklists and diet histories. The preliminary BPGs and a proposed list of different dietary assessment tools’ strengths and weaknesses (DATs-S&W) were generated from the literature. Following review by the expert group, they were presented as eight main questions comprising 47 guidelines.

### Participants

A list of experts was drawn up by the DIET@NET experts and research team to include nutritional epidemiologists, statisticians and public health specialists. The list of experts included authors of key publications on assessing dietary intake and those generated through a separate exercise undertaken to identify DAT for the Nutritools website, the DIET@NET review of systematic reviews of dietary assessment [15]. In addition, authors of nutritional epidemiology textbooks, lead speakers at relevant conferences and a panel of international experts on the DIET@NET Advisory Group were included. The participants did not know the identities of the other individuals in the group, nor were they informed of the specific answers of any individual.

### Data collection

In the first round, experts were invited by email to be involved in the development of the BPGs. If the experts accepted, they were emailed a consent form to complete. Ethical approval was not required in this study, because we were seeking opinions from experts.

The guidelines were sent by email as either two Word documents (BPGs and DATs-S&W) or as an embedded link to a Bristol Online Survey (University of Bristol, UK), depending on participant preference. Participants were asked whether each of the guidelines should be included or excluded, to provide suggestions on how to improve the guidelines and to give justification for any exclusion(s). Similar criteria were used in the second round, when participants were also asked to rate included guidelines as either Essential or Desirable.

Data collection occurred between 16 July until 25 August 2015 for the first round, and for the second round from 22 October to the end of November 2015. To increase the response rate, a reminder email was sent to emphasise the importance of completing the whole Delphi process. See Additional file 1: Section S1 for the data collection tool emailed for Delphi I.

The generation of consensus was performed by means of email exchange among the DIET@NET project expert group during Delphi rounds I and II. The experts were informed of the Delphi group’s collective response anonymously in each round.

### Generating a consensus

A consensus was pre-defined, following the recommendations of Sinha et al. [16, 17]. This was set at achievement of a 70% inclusion rating, i.e. less than 30% of participants rated the guideline as ‘exclude’ for each guideline in the first Delphi round. Due to the agreement usually improving in the second Delphi round [17], each guideline then had to achieve a 90% rating as either Essential or Desirable in the second round to be included. Of these, the guidelines achieving > 70% essential were defined as ‘essential guidelines’ whilst those achieving lower scores were defined as ‘desirable guidelines’.

Issues raised by the Delphi participants, such as suggestions for new wording of the guidelines, were reviewed by the DIET@NET expert group. After each round, they received an anonymous summary of all participants’ comments and feedback together with the level of agreement. They reviewed the tentative guidelines and made changes by rewording, combining, splitting or moving some guidelines. The strengths and weaknesses of the dietary assessment methods were reviewed by the DIET@NET experts.

## Results

### Participants’ characteristics from Delphi rounds

**Fig. 2:**
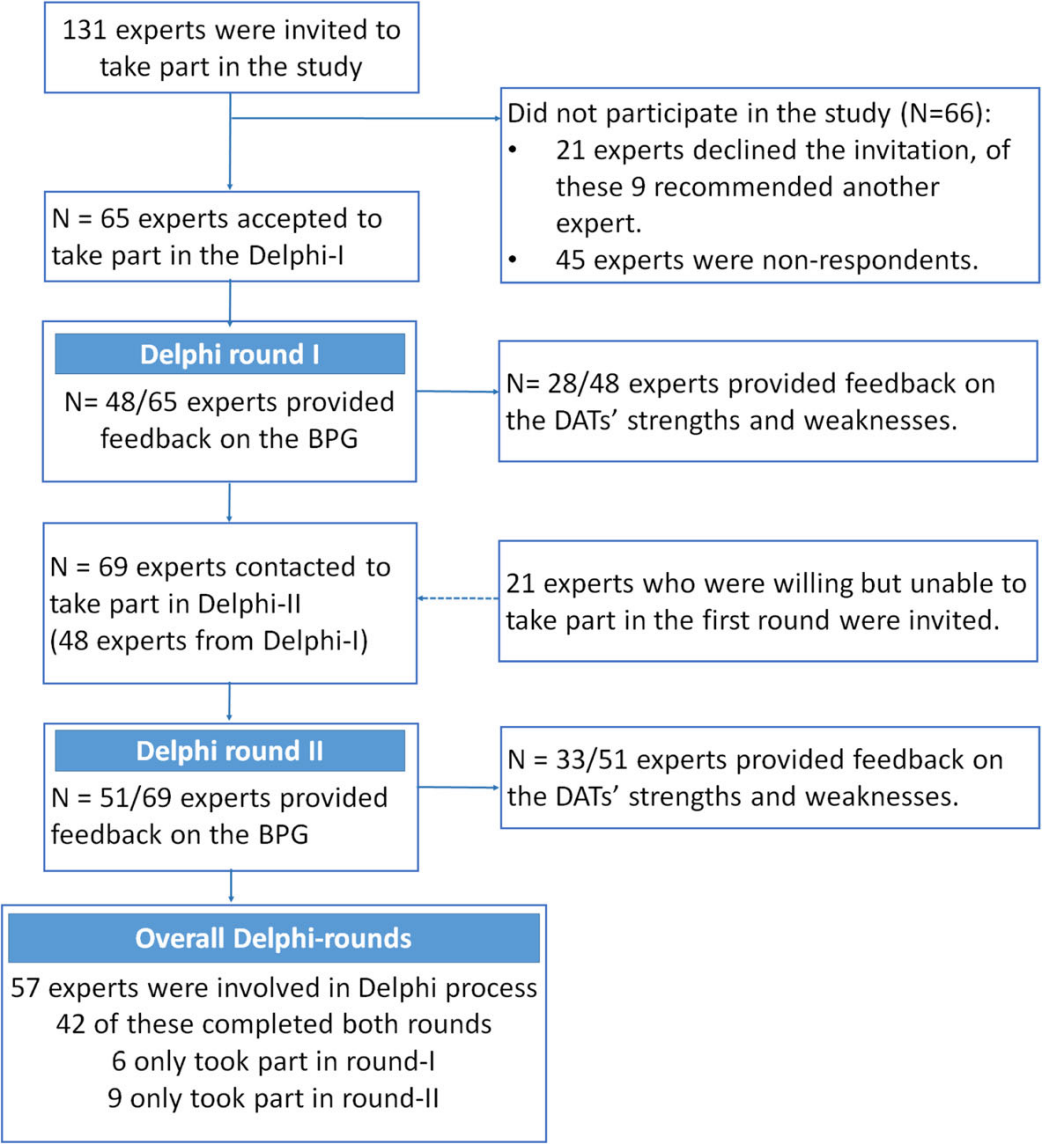
Experts of the Delphi consultation rounds

Overall 57 experts were involved in the Delphi rounds (listed in the Acknowledgements): 42 (74%) completed both rounds, 6 (11%) completed only the first round and 9 (16%) completed only the second round (Fig. 2). A total of 28 (58%) experts in the first round and 33 (48%) experts in the second round provided feedback on the DATs-S&W. Overall, 47 of the 57 (83%) participants were female. The Delphi participants came from a range of countries, mainly the UK, the USA and Australia (Table 1).

**Table 1:** Geographical spread of experts who participated in Delphi rounds

Country	Delphi I (*n* = 48), *n* (%)	Delphi II (*n* = 51), *n* (%)
UK	19 (39%)	20 (39%)
USA	7 (14%)	6 (11%)
Australia	6 (12%)	5 (9%)
Canada	2 (4%)	4 (7%)
France	2 (4%)	3 (5%)
Brazil	2 (4%)	2 (4%)
Netherlands	2 (4%)	2 (4%)
Italy	2 (4%)	2 (4%)
Belgium	1 (2%)	1 (2%)
Japan	1 (2%)	1 (2%)
Norway	1 (2%)	1 (2%)
Spain	1 (2%)	1 (2%)
Greece	1 (2%)	1 (2%)
New Zealand	1 (2%)	1 (2%)
Serbia	0 (0%)	1 (2%)

### Consensus on the Best Practice Guidelines

There were 47 tentative guidelines in the first Delphi round. Most of the changes between rounds 1 and 2 related to wording alterations or splitting, combining or moving guidelines, with one new guideline added to the second round based on feedback. The overall agreement in the second round was improved for 26 of the 48 guidelines to achieve an overall agreement ranging between 84% and 100%. Guidelines with less than 90% overall agreement were removed, resulting in 43 BPGs post Delphi II. Merging similar concepts whilst developing the final guidelines by the DIET@NET experts resulted in a set of guidelines grouped into four stages with 8 main guidelines split into 24 specific guidelines (19 Essential and 5 Desirable). A further 11 statements were included for 4 of the final guidelines; these clarified or extended the guideline and were included in the original Delphi rounds. These were included as explanations below the specific guidelines on how to evaluate published validation studies (guideline 5.1); the quality of the validation study (guideline 5.2); understanding the strength of agreement between methods (guideline 5.3); and how to decide if an existing tool could be improved (guideline 6.1). These were not rated as Essential or Desirable in the final guidelines because they were intended to provide additional explanation rather than being stand alone items.

### Best Practice Guidelines

The resulting BPGs include initial guidance to consider the study objective and purpose, followed by the main guidelines (Table 2 and Additional file 1: Figure S1) to consider when choosing which tool to use for assessing dietary intake. The strengths and weaknesses of each of the dietary assessment methods were also compiled (Additional file 1: Table S1).

**Table 2:** Best Practice Guidelines for dietary assessment in health research

E/D^a^	Stage I. Define what you want to measure in terms of dietary intake: the key a priori considerations to guide your choice of the appropriate type of dietary assessment tool (DAT)	
	*1*	*What? — Characteristics of the main dietary component of interest*
E	1.1	Clearly define what needs to be measured (e.g. intake of energy, food groups, specific or a range of macro- or micro-nutrients)
E	1.2	Determine how the dietary data will be analysed and presented (e.g. total daily or meal level intakes, food groups or nutrients)
	*2*	*Who? — Considerations around the characteristics of study participants*
E	2.1	Define the target sample in terms of characteristics (e.g. life stage, ethnicity, health status, body mass index (BMI), socio-economic level, country/region and setting — home, school, hospital)
E	2.2	Identify other issues that could affect the choice of DAT (e.g. literacy, numeracy, language, cultural, disability, time or familiarity with technology)
E	2.3	Consider the study sample size required in relation to the level of variation of your dietary component of interest and study power
	*3*	*When? — Time frame considerations*
E	3.1	Are you interested in ‘actual’/short-term (hours or several days, up to one week) or ‘usual’/long-term intake (e.g. months or years)? Consider what reference period (e.g. daily, weekly, monthly, yearly) would be best suited to your dietary component of interest
E	3.2	Will data collection in your study be retrospective or prospective?
	Stage II. Investigate the different types of DATs and their suitability for your research question	
	*4*	*Consider and appraise the different DAT types*
E	4.1	In relation to your research question, consider the suitability, strengths and weaknesses of different DAT types^b^
E	4.2	Think about participant burden (e.g. study participants’ potential willingness, time, ability, ethical considerations, interest in using different tools and access issues associated with different DATs)
E	4.3	Identify the availability of resources (e.g. staff skill, time, finances)
	Stage III. Evaluate existing tools to select the most appropriate DAT	
	*5*	*Research and evaluate available tools of interest*
E	5.1	Read any available published validation studies:
		• Has the DAT been evaluated to measure the dietary component you are interested in?
		• Has the DAT been evaluated in a population similar to your population of interest?
		• Is the nutrient database used appropriate?
		• Are the portion sizes used relevant?
D	5.2	Assess the quality of validation in terms of:
		• Has the DAT been compared to an objective method (e.g. biomarkers)?
		• Has the DAT been compared to a subjective method (e.g. a different self-reported diet assessment)?
		• What were the limitations of the validation study?
D	5.3	The strength of agreement between the two methods:
		• Is there any evidence of bias; do the methods agree on average?
		• Is there any evidence of imprecision; how closely do the methods agree for an individual?
	*6*	*If, based on the validation studies, none of the existing DATs is entirely or wholly suitable, consider the need to modify or update an existing DAT, or create a new DAT and evaluate it*
E	6.1	Decide whether an existing tool can be improved. Investigate whether:
		• Foods and portion sizes included are characteristic of your target population, and frequency categories are appropriate
		• The time period that the questionnaire refers to could be modified to better suit your needs
D	6.2	Consider the face validity of existing tools. Is there evidence the tool has been used to measure dietary intake in your population of interest?
D	6.3	Updated or modified tools may require re-evaluation. Consider if validation can be integrated into your study
	Select your DAT	
	Stage IV. Think through the implementation of your chosen DATs	
	*7*	*Consider issues relating to the chosen DAT and the measurement of your dietary component of interest*
E	7.1	Obtain information regarding DAT logistics (e.g. tool manual, relevant documents and other requirements from the DAT developer)
E	7.2	Check that the chosen DAT has the most appropriate food/nutrient database and software
E	7.3	Check the requirements for dietary data collection (e.g. entry, coding and software)
D	7.4	Consider collecting additional related data (e.g. was intake typical, supplement use)
	*8*	*Prepare an implementation plan to reduce potential biases when using your chosen DAT*
E	8.1	Consider potential sampling/selection bias and track non-participation/dropout/withdrawal at different stages
E	8.2	Minimise interviewer bias (e.g. ensure staff qualifications and training are appropriate, develop standardised training protocols and monitoring procedures)
E	8.3	Minimise respondent biases (e.g. use prompts, clear instructions)
E	8.4	Quantify misreporting

^a^Guidelines which achieved > 70% as essential were defined as Essential guidelines (*E*), whilst those achieving lower scores were defined as Desirable guidelines (*D*)

^b^See Additional file 1: Table S1 for DATs’ strengths and weaknesses

#### Pre-study: what is your research objective?

The purpose of the study has to be clearly defined, as this will determine the level of precision required for the DAT, the sample size and other aspects of the study design. With this in mind, the following sections describe each of the guidelines for each stage, and mark them as Essential or Desirable.

### Stage I. Define what you want to measure in terms of dietary intake: the key a priori considerations to guide your choice of the appropriate type of dietary assessment tool (DAT)

#### 1 What? — characteristics of the main dietary component of interest

##### 1.1 Clearly define what needs to be measured (e.g. intake of energy, food groups, specific or a range of macro- or micro-nutrients) (Essential)

Diet is usually described in terms of nutrient content, the food type or food group or dietary pattern [18]; consider which of these you need in your study. Some foods and nutrients are assessed more accurately than others. Foods consumed regularly are easier to report than infrequently consumed items [19]; FFQs or recalls/food diaries may be equally suitable. However, FFQs may not be extensive enough to capture infrequently consumed foods, unless they are specifically developed for the purpose. Estimates of food and nutrient intake involve random error (e.g. due to inaccurate food tables, limited days of recall affected by day-to-day variation) and systematic bias [20] (such as limited food tables and reporting bias; e.g. low energy reporters tend to under-estimate foods high in fats and sugars [21]).

A clear definition is needed of what is to be measured and the level of detail required; e.g. for energy intake the whole diet needs to be assessed, but for nutrients concentrated in some foods, an assessment of specific food may be sufficient.

##### 1.2 Determine how the dietary data will be analysed and presented (e.g. total daily or meal level intakes, food groups or nutrients) (Essential)

If the aim is to assess nutrient intake over the whole day or complete eating patterns, a more detailed and extensive DAT will be required than if people’s specific eating behaviours, such as breakfast consumption, snacks or skipping meals, need to be recorded. For the latter, some brief questions may suffice. Collecting information regarding portion size and number of daily servings allows for detailed food and nutrient analysis.

#### 2 Who? — considerations around the characteristics of study participants

##### 2.1 Define the target sample in terms of characteristics (e.g. life stage, ethnicity, health status, body mass index (BMI), socio-economic level, country/region and setting — home, school, hospital) (Essential)

The target sample needs to be defined in terms of their age, ethnicity, BMI and other characteristics. It is important to assess whether the participant can self-report dietary intake or whether a parent/proxy will be required. Assessing diet among young children or adolescents requires different methods due to their cognitive ability to report diet, as well as their motivation [22–24]. Dietary recall relies on memory, which is subject to a variety of errors [3]. Assessing diet in different ethnic groups may require different DATs that measure specific foods. However, using different tools for different ethnic groups presents a barrier to harmonisation across studies [25]. The use of dietary records or recalls allows for a range of different eating patterns to be recorded, unlike the fixed food lists of an FFQ. Consideration also needs to be made around customary portion sizes by age and sex when developing ethnic-specific DATs [26].

Participants with a low level of education, lower socio-economic status, those with a high BMI and smokers are more likely to under-report intakes than others [27]. Additional support may be required for these groups with regard to self-reporting of diet.

##### 2.2 Identify other issues that could affect the choice of DAT (e.g. literacy, numeracy, language, cultural, disability, time or familiarity with technology) (Essential)

A DAT needs to be usable by the study population. One of the issues that may arise when selecting a DAT is the time taken to complete the DAT, which can affect response rates and the completeness of collected information. For the 24HR, an interviewer with appropriate skills is usually required, unless new online systems (e.g. myfood24, https://www.myfood24.org; Intake24, https://intake24.co.uk; ASA24, https://epi.grants.cancer.gov/asa24/; food4me, http://www.food4me.org; EPIC-FFQ, http://www.srl.cam.ac.uk/epic/epicffq/) are being used. Food diaries require considerable literacy and organisation to complete, whilst for FFQs some mathematical ability to estimate frequency is needed, particularly for less common food items. Use of new technology to assess dietary intake is promising for children, adolescents and adults, as it can be faster and easier than the traditional methods [28]. However, some level of technological literacy and numeracy is required, which might hinder use among older adults and participants with a low literacy level. The impact of these issues in terms of measurement error should be considered.

##### 2.3 Consider the study sample size required in relation to the level of variation of your dietary component of interest and study power (Essential)

The sample size will depend on the characteristics of the dietary component to be measured. It needs to be large enough to provide precise estimates and have sufficient statistical power to detect any effects or associations of interest. Intra-individual variation (day-to-day variation in amount and in type of food consumed) and inter-individual variation (variations between persons in their usual nutrient intake) differ for particular foods and nutrients. Nutrients with lower day-to-day variation (e.g. protein) are likely to require fewer days of diet recording compared to nutrients concentrated in certain foods (e.g. vitamin A) [29]. For most nutrients, random day-to-day variation in intake within individuals is larger than the variation between individuals; these random errors affect study precision. Increasing the number of measurement days with 24HRs or food diaries will reduce to some extent the effect of within-individual variance on mean daily intake and increase the precision of the mean estimate [30]. Short-term measurements should be adjusted for random error if distributions of intake are needed [31]. Foods that are consumed episodically will require more days of intake assessment (see Section 3 below) or a larger sample size to obtain an appropriate estimate. If you are unsure how to calculate sample size, consult a statistician.

#### 3 When? — time frame considerations

##### 3.1 Are you interested in ‘actual’/short-term (hours or several days, up to one week) or ‘usual’/long-term intake (e.g. Months or years)? consider what reference period (e.g. Daily, weekly, monthly, yearly) would be best suited to your dietary component of interest (essential)

Long-term average intake or the usual/habitual pattern differs from intake reported for a single day or a few days. Short-term intake (e.g. over a single day), such as a 24HR, only represents a snapshot in time but may provide less biased dietary data [30]. More than one day of dietary information is preferred to estimate usual intake [32], since it can assess both within- and between-person variation. Nevertheless, to assess a mean population-level food or nutrient intake, one day of intake on a large sample will be adequate. Non-consecutive days may capture more individual variability [33]. A single 24HR may not be sufficient to describe participants’ long-term usual intake, in part because of social desirability bias and also day-to-day variation with reports of energy intake being higher on a weekend than on a weekday. Two recalls were found to be better than one, and three minimised the mean difference between reported and objectively measured intakes [34]. However, self-report has been challenged in terms of suitability for measurement of energy intake [10], although it may be useful for adjustment of other nutrients to improve risk estimates.

Prospective cohort studies tend to use FFQs, which can minimise day-to-day variation by assessing long-term dietary intake [35]. This approach was the only realistic option for large-scale epidemiological studies for a long time. However, FFQs are prone to considerable misreporting. Misreporting can occur for a range of reasons, including misunderstanding of frequency categories and the mathematics required to complete the questionnaire; grouping of food types; and over-reporting of foods considered healthful and under-reporting of options considered less healthful. New technologies now make it feasible to use more detailed methods such as 24HRs or food diaries [28] in cohort studies.

Statistical modelling may mitigate some limitations of having only a few days of intake (short-term methods) when assessing usual intake [22, 36]. Specifying the period during which dietary assessment takes place is important. Many FFQs use 6 months or the preceding year as a reference period to address seasonal variation in diet. Recruiting over 12 months will enable variation in intake during the year to be taken into account. Assessing diet at a specific time of year when a certain fruit is widely available could misrepresent usual micronutrient intake [30].

##### 3.2 Will data collection in your study be retrospective or prospective? (essential)

Cross-sectional surveys are used to obtain a ‘snapshot’ of the diet of a population. A range of DATs may be suitable; for example, a 4-day prospective diary is used in the National Diet and Nutrition Survey in the UK [37]. However, local surveillance of diet for public health may use retrospective FFQs [38] due to lower cost and ease of implementation.

In case-control studies, DATs that focus on current intake, such as 24HRs or food records, are not suitable, because information is needed about diet before the onset of disease. An FFQ or diet history probing details prior to disease onset will be the only possible tools for this purpose. In prospective (cohort) studies, dietary status at baseline is measured and related to later incidence of disease. In such studies, retrospective and prospective DATs, including multiple 24HRs, records, diet history and FFQs, have all been used successfully [1]. Cost and other logistical issues often favour using an FFQ in large longitudinal studies. However, using new technology may make it feasible to overcome these issues [4].

### Stage II. Investigate the different types of DATs and their suitability for your research question

#### 4 Consider and appraise the different DAT types

##### 4.1 in relation to your research question, consider the suitability, strengths and weaknesses of different DAT types (essential)

If you are new to dietary assessment methods, explore each DAT’s profile (Additional file 1: Table S1) to learn about the different DATs: food diaries, recalls, questionnaires, screeners and diet history. Each has distinct features and strengths and weaknesses. Then evaluate the suitability of using each method based on your research questions and study target group.

##### 4.2 Think about participant burden (e.g. study participants’ potential willingness, time, ability, ethical considerations, interest in using different tools and access issues associated with different DATs) (Essential)

Reducing participant burden may be important, such as when high levels of literacy or motivation are not possible or to ensure high participation rates and reduce attrition. One method of reducing burden relates to portion size estimation. Foods need not be weighed, but portions could be estimated either in food diaries or 24HRs, using age-specific food photographs or described in household measures [39]. With interviewers, respondents may be concerned that researchers will judge their reported dietary intakes, respondents may over-report ‘healthful’ foods and under-report ‘unhealthful’ foods [5], affecting the quality of dietary data [40]. Lack of motivation and cooperation among adolescents may hinder dietary assessment among this group [41] compared with children and adults [42].

##### 4.3 identify the availability of resources (e.g. Staff skill, time, finances) (essential)

It is important to consider the level of training and/or expertise required by staff to implement and analyse the selected DAT. Adequate training of the field researcher will help to produce reliable dietary intake measurements. Manual coding of recalls/diaries is expensive and time-consuming.

### Stage III. Evaluate existing tools to select the most appropriate DAT

#### 5 Research and evaluate available tools of interest

##### 5.1 Read any available published validation studies (essential)

- *Has the DAT been evaluated to measure the dietary component you are interested in?*
  When possible, validated DATs should be used; however, validation should be relevant to the foods/nutrients of interest. It is important to check how well the DAT performed in the validation study for the food or nutrient of interest.
  The validity of a DAT will depend on accurate estimation of frequency and portion sizes, on the quality of the nutrient database and in the collection of data [43]. Measurement of absolute validity is difficult to establish, requiring the comparison method to be an objective measure such as recovery biomarkers, e.g. doubly labelled water. Relative validity (the comparison of two instruments of the same kind [30]), through use of multiple DATs, is more commonly used to detect bias [44].
- *Has the DAT been evaluated in a population similar to your population of interest?*
  Determine whether validation studies support the use of the candidate DAT for your study population. Population characteristics/covariates to be considered are life stage, ethnicity, cultural differences in diets, geographical area, education/literacy, age range, sex, types of diets and relevance of foods consumed at the time the DAT was validated [45, 46].
- *Is the nutrient database used appropriate?*
  The nutrient database used should be appropriate, comprehensive and up to date for the study population. Limited coverage of foods in the database, missing nutrient data, differences in software packages, incompatibility of databases [45], recipe, portion size allocations and bias in variability in recipes should be considered. This may be more difficult for processed foods due to the complexity of the food market and its rapid changes; most nutrient databases do not capture data on food reformulation. Composite dishes, either purchased or homemade, can vary due to differences in recipes. Weighing recipe ingredients is more practical than chemical analysis [47]. Standardised calculation procedures should take into account weight loss during cooking and nutrient losses into cooking water [48]. Nutrient retention factors may be applied to calculate the nutrient composition of a cooked food from the uncooked food [49]. Limitations and gaps in food composition tables need to be considered for coverage of nutrients. For example, total fibre is available in most food composition tables, but results differ according to the chemical analyses method used [50]. Sub-components of fibre, such as soluble and insoluble fibre, may not be available.
- *Are the portion sizes used relevant?*
  Accurate estimation of food portion sizes is important; errors are often introduced due to incorrect portion size quantification or use of an ‘average’ portion size [51]. Food photographs or food models can be provided; however, they only provide a limited number of foods and food portion sizes [52]. Portion size measurements should be relevant to the study population, characteristics and life stage. The type of food will influence reliability of portion size estimation; pre-packaged foods will have a weight declared which could be recorded. Participants’ perception of portion sizes from photographs or ability to conceptualise amounts along with memory limitations will affect the precision of portion size recording [51].

##### 5.2 Assess the quality of validation in terms of: (desirable)

- *Has the DAT been compared to an objective method (e.g. biomarkers)?*
  Objective methods to assess nutrients include clinical indicators or biomarkers [53], which vary in response to intake [30]. Biomarkers can reflect intake over the short term (past hours/days), medium term (weeks/months) and long term (months/years), depending on the sample type, e.g. blood, hair [8]. Ideally all DATs should be validated against an objective measure of intake. Recovery biomarkers such as 24-hour urine nitrogen and potassium excretion and doubly labelled water reflect absolute nutrient intake over a short time [54]. These are the best approaches to use for absolute validity of the tool. Predictive biomarkers (e.g. urinary fructose, sucrose and dietary sugars) have a lower overall recovery, and concentration biomarkers (e.g. serum carotenoids) correlate with dietary intake [55, 56]. Predictive biomarkers may be useful for validation studies; however, since concentration biomarkers cannot be translated into absolute levels of intake, they are less reliable for validation studies. Concentration biomarkers may be used for estimation of diet-disease risk associations as a substitute for or as complementary to dietary assessments [57]. Recovery biomarkers provide an estimate of absolute intakes as they are based on the concept of the metabolic balance between intake and excretion over a period of time, but only a few are known [58].
- *Has the DAT been compared to a subjective method (e.g. a different self-reported diet assessment)?*
  Although comparison with an objective method is preferable in terms of assessment of validity, this may not be available since these studies are costly and difficult to undertake. Comparison with an alternative form of dietary assessment is referred to as ‘relative validity’. However, comparison of one DAT against another risks correlated error between dietary assessment methods [30]. Any new dietary assessment should be compared against a more established method with greater face validity [59].
  It should be noted that the 7-day weighed food record was regarded as the ‘gold standard’ until studies that validated weighed food records with doubly labelled water found high levels of under-reporting [30]. Despite this, food records have been used as a standard to gain an insight into regular food intake [60], and they are often regarded as the most precise method for estimating food or nutrient intake [61].
  In addition to validity, test-retest reliability or reproducibility may also be relevant where diet is being measured at multiple time points.
- *What were the limitations of the validation study?*
  The comparison DAT used in the validation study also needs to be assessed in terms of scope, the time frame/number of days, the main type of measurement error, memory requirements and also an assessment of cognitive difficulty. For an FFQ that is being validated, the agreement with an alternative method will be higher if multiple days of reference data have been collected. Furthermore, to measure within-individual variability, 2 or more days of dietary intake are required, from at least a sub-set of the population [30].
  When considering a validation study, it may be helpful to use a scoring system [14]. The authors in the study by Serra-Majem et al. [14] have developed a scoring system (0 = poorest quality to 7 = highest quality) for validation studies. This was based on the sample size, the statistics used, the data collected, seasonality and the inclusion of supplement measures. The authors identified issues relating to the poor quality of validation research: inadequate description of study details such as the respondent characteristics; design of the questionnaire; and adequacy of the reference data. Studies which reported relative validity, i.e. comparing two self-reported measures of diet, scored less than those which compared a self-report with a biomarker.

##### 5.3 The strength of agreement between the two methods (desirable)

- *Is there any evidence of bias; do the methods agree on average?*
  Consider the extent to which a DAT under- or over-estimates dietary intake compared to another, possibly better DAT. This can be described using the Bland-Altman technique [62] for method comparison. The mean difference of the two methods of measurement is plotted against the average. Errors associated with dietary intake may be correlated, and this can lead to overinflated agreement between methods. In general, the use of correlation as a method of comparison is not recommended, since it does not measure agreement between methods. Other statistical tests are also used in dietary assessment method validation [63], for example, the method of triads [64], which evaluates the association between three measurements: the test method, the reference method and a biomarker. This method calculates the validity coefficient between the observed and ‘true’ dietary intake and assumes a linear correlation between the three variables, for example, validating an FFQ (measuring carotenoid and vitamin E intake) using weighed food records and plasma biomarkers [65]. This method has limitations, whereby it is possible to generate validity coefficients greater than one [66].
- *Is there any evidence of imprecision; how closely do the methods agree for an individual?*
  Precision provides a measure of the closeness of two methods for estimating diet for the individual [30], assessed over the whole sample. A DAT is considered precise if the estimated intake from the tool is close to the estimate from the reference tool, taking account of bias. The Bland-Altman technique also assesses precision with limits of agreement between the two DATs.

#### 6 If, based on the validation studies, none of the existing DATs is entirely or wholly suitable, consider the need to modify or update an existing DAT, or create a new DAT and evaluate it

##### 6.1 Decide whether an existing tool can be improved. Investigate whether: (Essential)

- *Foods and portion sizes included are characteristic of your target population, and frequency categories are appropriate*
  Food consumption patterns change over time, influenced by income and socio-cultural preferences [67]. The DAT selected should be applicable to the population of the study. Investigate whether the food list and portion sizes used in the DAT are current.
- *The time period that the questionnaire refers to could be modified to better suit your needs*
  Alteration of the time period the FFQ measures must be done with caution, as this could affect the validity of the tool and may require the FFQ to be revalidated. As one example, if an FFQ assesses the diet for 3 months, it could be converted to 12 months if the study was focusing on a nutrient that has seasonal variability.

##### 6.2 Consider the face validity of existing tools. Is there evidence the tool has been used to measure dietary intake in your population of interest? (desirable)

Face validity indicates whether food or nutrient intake results are sensible for your population [68]. It is important to check face validity to ensure usability and adequate response rate. For instance, the face validity of a food intake questionnaire was obtained by comparison with the opinions of practising UK Registered Dietitians. In this questionnaire, foods included were considered representative of general dietary advice [69]. Make sure that the language, format and procedures are understandable to your population of interest.

##### 6.3 Updated or modified tools may require re-evaluation. Consider if validation can be integrated into your study (desirable)

If you plan to update or modify the DAT, such as the food list or food portion sizes, then the tool should ideally be re-evaluated.

New tools will also require validation. To facilitate the construction of new tools, the Nutritools website will enable the creation of new online questionnaires with database mapping through the food questionnaire creator.

When designing a new FFQ, obtain lists and portion sizes of the most important foods and the percentage of foods contributing to nutrients of interest in your population, for example, from national surveys [70]. It is also important to consider factors that may affect the validity of a DAT. For an FFQ, these can be respondent characteristics (e.g. literacy); grouping of foods on the FFQ; frequency categories and time frame; and quality control of data management (e.g. reduce coding errors by setting limits on data entry and validation rules) [59].

### Next step: select your DAT

The selection of the DAT will depend upon the answers to the previous questions; whether it is to capture regular eating patterns (e.g. FFQ or repeated 24HR) or recent food consumed (e.g. diet record or 24HR) and the study design.

### Stage IV. Think through the implementation of your chosen DATs

#### 7 Consider issues relating to the chosen DAT and the measurement of your dietary component of interest

##### 7.1 Obtain information regarding DAT logistics (e.g. Tool manual, relevant documents and other requirements from the DAT developer) (essential)

The researcher may have to contact the DAT owner to obtain relevant documents for using the DAT. Other requirements from the DAT developer may be a contract agreement between the tool owner and the researcher, payment, or an acknowledgement.

##### 7.2 Check that the chosen DAT has the most appropriate food/nutrient database and software (essential)

An important pre-condition in selection of a DAT is an up-to-date, relevant nutrient database [71]. Nutrient databases may be incomplete for some nutrients. Evaluate which year the nutrient database refers to and whether there have been any updates. Nutrient databases tend to be ‘out of date’ [72], and among their limitations are the partial or limited coverage of nutrients and analytical limitations [45], which are influenced by seasonal variations and regional disparities [73].

Although dietary assessment software and innovative technologies in DATs can reduce researcher and respondent burden, it is still difficult to avoid measurement error completely, in part due to embedded nutrient tables [74].

##### 7.3 Check the requirements for dietary data collection (e.g. Entry, coding and software) (essential)

It is crucial to check the requirements for dietary data entry. One issue is how recipes are handled in the computer program, making allowances for losses of water and vitamins during cooking [75]. Entering recipes and consideration of raw to cooked values in databases, particularly in low- and middle-income countries, is another important issue that can be overlooked and can lead to substantial error [76, 77].

Appropriate software is necessary to link each food item recorded to the nutrient database when coding large amounts of data [5]. Use of new technology in measuring dietary intake has the potential to reduce respondent and researcher burden, automating data processing and enhancing participants’ willingness to report their dietary intake [78]. The incorporation of quality-control procedures at each stage of the dietary assessment method; training sessions for interviewers and coders; standardisation of interviewing techniques and questionnaires; and pre-testing and piloting the questionnaire will minimise systematic errors [30, 79].

##### 7.4 Consider collecting additional related data (e.g. was intake typical, supplement use) (desirable)

For acute measures of diet (e.g. 24HRs) participants should ideally be asked if the day of recording was typical and, if not, why not. DATs may gather additional information on dietary supplement intake [5]. This is important in low- or middle-income countries, where micronutrient deficiencies prevail and where provision of supplements (vitamin A, iron, folic acid) is common [80]. Depending on the study question, information on how foods are prepared or stored, and additional details on the type of food that was consumed (e.g. whole-grain, sugar-free or a fat-free food item) could be useful.

#### 8 Prepare an implementation plan to reduce potential biases when using your chosen DAT

##### 8.1 Consider potential sampling/selection bias and track non-participation/dropout/withdrawal at different stages (essential)

Researchers should minimise selection bias and non-response bias using an appropriate sample size from the target population ensuring that participants are representative of the wider population. Engaging the interest of participants prior to the study may prevent dropouts [81] that can affect the generalisability of findings.

##### 8.2 Minimise interviewer bias (e.g. ensure staff qualifications and training are appropriate, develop standardised training protocols and monitoring procedures) (essential)

If you decide to interview participants, appropriate training of staff will reduce interviewer bias. Interviewers need knowledge to correctly identify, describe and check foods and to be consistent with all participants. Question wording, probing questions and an ability to establish a good relationship with the respondent can all influence the quality of the data collected. Records should be reviewed with the respondent in order to clarify food entries and to probe if foods have been forgotten [82]. Field interviewers should have knowledge of the foods, customs and language of the study population [83].

##### 8.3 Minimise respondent biases (e.g. use prompts, clear instructions) (essential)

Social desirability bias is common [84]. Under-reporters tend to be selective, by reporting fewer servings from food groups with higher energy densities [85]. Prompt questions and reminders can be included to minimise likely omissions.

##### 8.4 Quantify misreporting (essential)

It is essential to identify and minimise potential misreporting. Misreporting is a complex problem in dietary assessment that comprises both under- and over-reporting and introduces error into the estimation of energy intake and nutrients [27]. A reasonable approach to identify under-reporters is the application of the Goldberg equation during analysis. However, note that the use of this method may also lead to bias or misclassification because of the assumptions used to estimate total energy expenditure [86]. Furthermore, recent guidance suggests that rather than excluding implausible energy reporters from the analysis, it should be stratified by reporting status [87]. We recommend trying to understand the reasons why participants under-report in each study, as it is known that all dietary assessment methods are prone to misreporting.

## Discussion

Dietary assessment is complex, and guidance on the selection of the most appropriate DATs is needed. The DIET@NET partnership has generated expert consensus on BPG for dietary assessment in health research, using the Delphi technique. The Delphi technique is an iterative process which allowed integration of expert opinions into BPGs with 8 main guidelines, 24 elaboration guidelines and 11 sub-elaboration guidelines.

Numerous subject experts were recruited. They had a good diversity of knowledge, from a range of different backgrounds and countries. This approach was a practical way of generating international consensus. Not all members from the Delphi group completed the first or second Delphi round, and similarly not all members from the Delphi group completed the embedded survey on the DATs-S&W, but in both cases there was a reasonable 50% response rate for the Delphi rounds and a 26% dropout rate. The final sample of experts is higher than most guidelines developed using face-to-face meetings or workshops [55].

Feedback and consensus can be problematic in the Delphi process. For this study, the collective opinions from the Delphi group were fed back to the DIET@NET experts. Delphi produces more robust findings and allows a higher degree of flexibility than other consensus methods (e.g. the nominal group technique or models developed by the National Institutes of Health [88]).

A strength of the Delphi technique is that it explores issues objectively, encouraging views free from peer group pressure, and allowing participation of larger groups [89]. The experts’ responses were reviewed by the DIET@NET experts. Subject anonymity is important, as this can reduce the effect of dominant participants, which is a concern when using group-based processes to collect information [90]. Another notable strength of the BPGs was the prioritisation of the guidelines as either Essential or Desirable.

These new guidelines on conducting research in nutritional epidemiology complement another recent guideline on reporting findings from nutritional epidemiology. The STrengthening the Reporting of OBservational studies in Epidemiology (STROBE-nut) guidelines [55] were developed similarly to the BPGs using a Delphi technique. Both sets of guidelines support improved conduct of research and reporting of results in nutritional epidemiology. In addition, this work will complement the new guidelines for assessing biodiverse foods in dietary intake surveys published by the Food and Agriculture Organization (FAO) [91].

The BPGs should be used when researchers are designing their study protocol. This paper provides only highlights regarding considerations for dietary assessment in epidemiology; further details and explanations can be found in additional resources such as the Nutritools website and the National Cancer Institute Dietary Assessment Primer [5]. The use of these guidelines cannot replace the need for appropriate validation studies or other study development work. However, where development of a new method is not practical, these expert-generated BPGs can provide consistency for selection of the most appropriate tool.

Selecting a suitable tool should lead to more accurate dietary assessments, better quality research and, consequently, more valid results. Practicalities including associated costs need to be considered when making the selection.

We anticipate that the BPGs will continue to evolve, with testing of the guidelines in field work scenarios allowing determination of their efficacy. The successful application of these BPGs will depend on the availability of validated DATs. The BPGs will be available online in interactive form on the Nutritools website (http://www.nutritools.org). Case studies will also be provided on the website as examples on how to use the BPGs in practice. The BPGs will also be promoted through the Enhancing the QUAlity and Transparency Of health Research (EQUATOR) Network [92]. We will be able to monitor use of the website and track reference to the guidelines in publications. Future work will allow comparison of studies using or not using the guidelines to evaluate effectiveness. In addition, the Nutritools site will provide access to a number of validated DATs. A linked food questionnaire creator will allow researchers to follow the BPGs to either adapt existing tools or create a new questionnaire if a suitable DAT is not available.

## Conclusions

In conclusion, BPGs have been developed, using expert feedback, to support researchers in their selection of the most appropriate DAT. We anticipate that researchers will use the BPGs primarily through the innovative Nutritools website. The use of these guidelines, together with the relevant validation studies for the DATs, should lead to better quality research.

## Additional file

Additional file 1: Section S1. The data collection tool for Delphi I data collection. **Figure S1**. Best Practice Guidelines diagram. **Table S1**. Description of dietary assessment tools (DATs) and their strengths and weaknesses. (DOCX 267 kb)

## Abbreviations

24HR: 24 hour recall
BPGs: Best Practice Guidelines
DAT: Dietary assessment tool
DATs-S&W: Dietary assessment tools’ strengths and weaknesses
DIET@NET: DIETary Assessment Tool NETwork
FFQ: Food frequency questionnaire
