# Supplementary material for: DIET@NET: Best Practice Guidelines for dietary assessment in health research
Source: BMC Med. 2017 Nov 15;15:202. doi: 10.1186/s12916-017-0962-x (PMC5686956; doi:10.1186/s12916-017-0962-x)
Supplement: Additional file 1: Section S1. — The data collection tool for Delphi I data collection. Figure S1. Best Practice Guidelines diagram. Table S1. Description of dietary assessment tools (DATs) and their strengths and weaknesses. (DOCX 267 kb) [file 12916_2017_962_MOESM1_ESM.docx]

# Appendix A.

DIET@Guidelines - Best Practice Guidelines for the collection and analysis of dietary data in population and clinical studies

Thank you for taking part in the DIET@Guidelines (Define, Investigate, Evaluate, Think through - Assessment Tool Guidelines) first Delphi round.

These Best Practice Guidelines are aimed at researchers and public health practitioners who are not experts in dietary assessment.  They are meant to be key considerations when designing and conducting studies that involve the collection and analysis of dietary data.

For each guideline, please could you indicate whether you feel it should be included or not by typing **Y** or **N** in the allocated column. 
If you select ‘N’, please can you **justify why** in the comment column.
Please also use the comment column to suggest any improvements to the guideline, for instance in terms of the wording, its positioning or to add a reference supporting your comment. You can also provide overall comments at the end of each stage.

If you do not type 'Y' or 'N', we will include that item. Guidelines with ≥70% agreement will be put through to the next Delphi round.

As per the invitation email you were sent, your comments will be treated anonymously with only the Project Manager, Research Support Assistant and Principal Investigator being able to monitor responses. 

The deadline for the Delphi round is **Monday 10 August 2015**.

Thank you very much for supporting this work and providing your expertise.

**The DIET@NET Partnership**
Investigators: Janet Cade (Principal Investigator), Nisreen Alwan, Paul Finglas, Tim Key, Barrie Margetts, Andy Ness, Sian Robinson, Toni Steer, Petra Wark
Project team: Katharine Greathead, Neil Hancock, Jozef Hooson

**Overview of the Guideline Stages**

*The diagram below gives an overview of the Best Practice Guidelines which takes researchers through 5 stages of considerations to help them choose the most appropriate Dietary Assessment Tool. There are 8 guidelines in all, many of which are broken down into subheadings.*

Revisit in light of Stage I

I. **D**efine your research question

Who?

What?

When?

II. **I**nvestigate Dietary Assessment Tool (DAT) types

Strengths & weaknesses

III. **E**valuate validity of available DATs

Validation robustness

Potential of non-validated tools

V. **T**hink through the implementation of your
 chosen tool

Dietary exposure measurement

Potential biases

Nutrient database selection

*IV. select your DAT*

Do you have any comments about the overview?

| **Stage I guidelines. Define your research question: key *a priori* considerations to guide your choice of the appropriate type of Dietary Assessment Tool (DAT).** | | | | |
| --- | --- | --- | --- | --- |
|  | **Include Y or N?** | | **Comment(s)** | |
| **1. What? - characteristics of the dietary exposure of interest.** | | | | |
| 1a. Clearly define **what** needs to be measured. *For example: energy intake; food groups; specific foods; episodically eaten foods; specific or a range of macro- or micro-nutrients; other food constituents (e.g. flavonoids); meals patterns, meal frequency and meal sizes; components needed to assess a priori dietary patterns.* |  | |  | |
| 1b. Consider the existing literature to ascertain the **level of variation** around your dietary exposure of interest in terms of frequency and range, and identify the drivers of the variation. *This may affect the type of tool that is suitable and the duration of intake data collection. Some micronutrient intakes can have higher day-to-day and week-to-week within-individual variation and use of vitamin supplements may need recording; the foods of interest may be infrequently consumed.* |  | |  | |
| 1c. Determine the **measurement level** required, whether group means, population means or individual intakes (*this informs the timescale of the DAT and number of times it might be used)* and establish the **level of change or difference** that needs detecting and sample size required *(tools vary in terms of their sensitivity).* |  | |  | |
| 1d. List **other dietary components** which need to be measured by the tool and accounted for in the statistical analysis as they may be potential confounders *(e.g. vitamin supplement use; eating events such as snacking; fat and vitamin A intake; vitamin C and fruit and vegetable intake; table salt use; timing of meal).* |  | |  | |
| 1e. Consider **previous research** on the measurement of the dietary exposure *(some nutrients such as sodium cannot be appropriately measured with a dietary assessment tool alone).* |  | |  | |
| **2. Who? – considerations around the characteristics of study subjects.** | | | | |
| 2a. Define the **sample** in terms of age range, sex, ethnicity and socio-economic group. |  | |  | |
| 2b. Identify literacy, numeracy, language, cultural, disability or motivation **issues** that could affect the dietary data collection method. |  | |  | |
| 2c. Consider the **generalisability** of the study sample (*e.g. characteristics of the sample may lower its external validity e.g. vegetarianism).* |  | |  | |
| 2d. Consider the **power** of the study sample required. *This may depend on the characteristics of the dietary exposure to be measured. The sample size needs to be large enough to provide precise estimates and detect any effects or associations of interest as being statistically significant.* |  | |  | |
| **3. When? – time frame considerations** | | | | |
| 3a. Establish whether **usual** (habitual) intake or **acute** (actual) intake will be measured and whether dietary data collection will be **retrospective** or **prospective**. |  | |  | |
| 3b. Determine the **reference period** (length of time) of retrospective recall/FFQ or prospective diary in light of the characteristics of the dietary exposure (as established though guideline 1) and the association being studied *(e.g. past week, month, year or number of days in the future; inclusion of weekend and all weekdays)*. |  | |  | |
| 3c. Consider the **timing** of dietary data collection linked to the key exposure of interest; for example in relation to diagnosis of disease or seasonality *(e.g. collection of vitamin D intake and comparison to status will be affected by season)*. |  | |  | |
| Do you have any overall comments on the Stage I guidelines? | | | | |
| **Stage II guidelines. Investigate DAT types and their suitability in view of your research question.** | | | | |
| **4. Consider and appraise the different DAT types.** | | | | |
| 4a. In relation to your research question, understand the main **strengths and weaknesses** (in terms of their scope and practical considerations) of different self-reported tool types  *(Delphi round participants: These strengths and weaknesses will presented as a table format which, due to its size, has been sent to you in Document 2 for you to review)*. |  | |  | |
| 4b. Identify any **practical limitations** in terms of the availability of staff, time and financial resources (or other) in light of DAT type requirements and sample size and consider how these might affect or potentially compromise your assessment. |  | |  | |
| 4c. Think about **participant burden**: your study subjects’ willingness, ability and interest in using different tool types in view of the usability, acceptability (e.g. length of time taken to complete) and access issues associated with different DATs. |  | |  | |
| 4d. **Re-evaluate DAT types** in light of your research question (what, who, when as per Stage I) and any practical constraints. |  | |  | |
| Do you have any overall comments on the Stage II guidelines? | | | | |
| **Stage III guidelines. Evaluate existing DATs to fine-tune your choice of the most appropriate DAT.** | | | | |
| **5. Research and evaluate available ‘validated’ tools of interest.** | | | | |
| 5a. Read the published **validation study** and consider its **relevance** in terms of: | | | | |
| - whether the tool is validated to measure the same dietary exposure as the one you are interested in; | |  | |  |
| - whether the energy and other intakes reported seem credible; | |  | |  |
| - whether it has been pre-tested and validated in a similar population *(consider differences in age range, sex, geographical area etc.)*, in a similar era and for a similar study design *(being aware of differences compared with the proposed study is important).* | |  | |  |
| 5b. Assess the **quality of validation** in terms of: | |  | |  |
| - the comparator used – whether it is a more detailed *DAT (e.g. 7-day weighed diary)* with measurement error that is uncorrelated with the test DAT (*e.g. test and comparator not both reliant on memory)* or a more objective gold standard method (valid biomarkers); | |  | |  |
| - whether the nutrient database used seems appropriate *(e.g. up-to-date, relevant geographical source)*; | |  | |  |
| - whether portion sizes were assessed and how (*e.g.* *‘average’ assigned, photographic food atlas or food models)* and whether protocols were followed; | |  | |  |
| - what the limitations of the validation study were *(e.g. sample size, time frame of tool administration and comparator – these may have already been identified by the study authors).* | |  | |  |
| 5c. Consider the **strength of the validation** results in terms of: | |  | |  |
| - whether the validation measures agreement (e.g. Bland-Altman method, intraclass correlation or kappa statistic based on category or ranking) or only correlation; | |  | |  |
| - how well the methods agree:   - are the Bland-Altman limits of agreement sufficiently narrow in light of the measurement level required for your dietary exposure of interest? | |  | |  |
| - - are Bland-Altman estimates of bias (the mean differences) sufficiently small in light of the measurement level required for your dietary exposure of interest? | |  | |  |
| - - how high is the intraclass correlation or kappa statistic? | |  | |  |
| - in the absence of a measure of agreement of absolute intake, do the methods agree in how they rank intake relative to other participants (kappa or weighted kappa statistic)? | |  | |  |
| - in the absence of any of measures of agreement, what is the strength of the correlation? | |  | |  |
| 5d. Consider the evidence of good **test-retest reliability/reproducibility** (over time, over different observers). | |  | |  |
| 5e. Re-evaluate the **suitability** of the identified tool in view of your dietary exposure of interest and population studied (as established in Stage I). | |  | |  |
| **6. Consider the need to potentially modify, update or create a new tool and re-validate it.** | | | | |
| 6a. Consider the **face validity** of the tool and whether there is evidence the tool has been used to measure dietary intake in your population of interest. | |  | |  |
| 6b. Decide on the potential need for adapting and improving **portion size estimation** to improve dietary data quality, using national surveys for age and sex portion size data. | |  | |  |
| 6c. For FFQs and checklists, investigate **adapting the food item list** so: | |  | |  |
| - foods of interest are included *(e.g. those with highest content of key exposure such as omega-3)*; | |  | |  |
| - vitamin supplementation is queried and matched to suitable database for micronutrient intake measurement; | |  | |  |
| - foods and frequencies are relevant to your population of interest *(e.g. in view of evidence from national surveys, nutrient databases or pilot study with open-ended recall or diary methods)* and non-contiguous response options are provided. | |  | |  |
| 6d. Updated or modified tools are likely to require **re-validation**, a resource intensive but worthwhile process given the ability to adjust for measurement error; consider how to integrate validation into your study. | |  | |  |
| Do you have any overall comments on the Stage III guidelines? | | | | |
| **Stage IV. Select your DAT.** | | | | |
| There are no guidelines for this section as this represents the moment of choice for the guideline user before proceeding to the final section (Stage V). | | | | |
| **Stage V Guidelines. Think through the implementation of your chosen DAT.** | | | | |
| **7. Consider issues relating to the measurement of your dietary exposure of interest.** | | | | |
| 7a. Ensure appropriate **number and timing of measurements** (week and weekend days covered) and length to follow-up (over a year, week, month, day, meal) and ensure the DAT is sensitive enough to detect dietary intake change over the time period. | |  | |  |
| 7b. Ensure **portion size estimation** which is appropriate for your population sample is integrated within the tool. | |  | |  |
| 7c. Obtain information on **tool logistics**: tool manual, relevant documents and other requirements from the tool developer. | |  | |  |
| 7d. If needed, collect **additional data** (e.g. illness, festivals) that may affect usual or acute intake, bearing in mind ethical considerations and the additional burden placed on participants. | |  | |  |
| 7e. Select the most **appropriate food/nutrient database,** evaluating potential limitations and sources of error in light of your research question – *completeness of dataset, whether it includes up-to-date reformulations (e.g. sodium); how database developers have dealt with missing foods and nutrients or aggregated foods that are pertinent to the research question; the ability to link individual ingredients as recipes; whether disaggregation of foods from composite dishes is possible (e.g. for total meat, fruit and vegetable intake).* | |  | |  |
| 7f. Evaluate and factor in dietary **data collection, entry and coding** requirements *e.g. staff numbers, skills and training, technological requirements, time.* | |  | |  |
| **8. Address the source and range of potential biases associated with the chosen DAT.** | | | | |
| 8a. Identify and minimise potential **sampling/selection** and **response biases**; track non-participation/dropout/withdrawal and non-compliance at different stages. | |  | |  |
| 8b. **Recall bias** for memory dependent DATs – consider whether self-reported intake or not; interview or online; multiple pass approach; use of protocols that are suitable for the sample (e.g. proxy rating for children with more than one carer e.g. parent, nursery or school). | |  | |  |
| 8c. **Interviewer bias** – ensure qualifications and training of staff are appropriate; put standardised protocols in place and set up monitoring procedures. | |  | |  |
| 8d. **Measurement error biases** - quantify and minimise misreporting *(important in validation of tools)*; additional data may need to be assessed *(e.g. weight, height, age, sex, physical activity to estimate BMR or energy expenditure (overweight people more likely to under-report), socio-economic status, education level, eating habits)*; repeated measures may be required. | |  | |  |
| Do you have any overall comments on the Stage V guidelines? | | | | |

Thank you for completing all five stages. Do you have any further comments on the Best Practice Guidelines as a whole?

On behalf of the DIET@NET project team and partners, **thank you very much** for taking the time to complete this first Delphi round and provide your expert feedback. We look forward to your participation in round 2.

We would be grateful if you could also comment on the table of strengths and weaknesses of different DAT types in the separate Word document sent to you.

Once again, many thanks for your contribution. If you have any questions about DIET@NET or the Best Practice Guidelines, please contact the DIET@NET project team.

Jozef Hooson – Research Support Assistant – [j.hooson@leeds.ac.uk](mailto:j.hooson@leeds.ac.uk)

Katharine Greathead – DIET@NET Project Manager – [k.e.m.greathead@leeds.ac.uk](mailto:k.e.m.greathead@leeds.ac.uk)

# Appendix B.

Best Practice Guidelines diagram


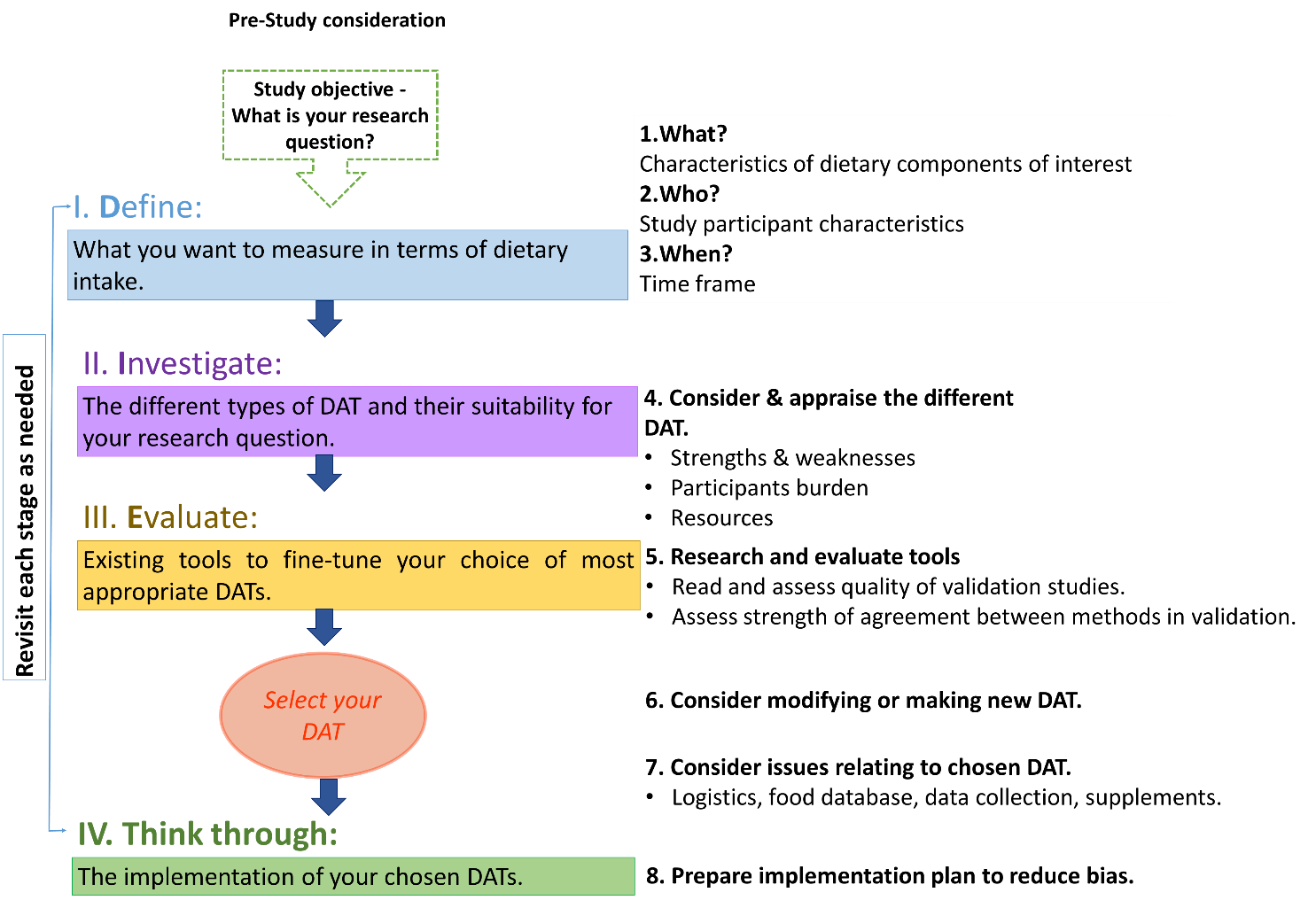


# Appendix C

Description of dietary assessment tools (DATs) and their strengths and weaknesses

| **Overall description** | **Strengths** | **Weaknesses** |
| --- | --- | --- |
| **FOOD DIARIES (FD)** | | |
| Prospective, short-term methods where details of all foods and drinks are recorded by the participant as they are consumed, usually over several days. Amount of food eaten can be either estimated using household measures (estimated food diary) or weighed by the respondent or research assistant in the home (weighed food diary). It can be long term method if carried out multiple times, i.e. over multiple phases. Also known as food records or diet records. Can be completed online (see emerging technologies section). | - Provides detailed data on all food and drink consumption and portion size description, leading to good estimates of short term total dietary intake and total nutrient intake if completed thoroughly and nutritional supplement use is assessed. - Allows collection of contextual information (e.g. meal timing, location, brand, eaten with whom, TV/computer/device etc.). - Provides food and nutrient data that can be used in numerous types of analysis to answer a variety of research questions. - Potentially little reliance on memory when completed prospectively i.e. when food is consumed or soon after. - Weighed food diaries - weight of ingredients, final cooked weight and food waste can be measured leading to detailed information of consumption and good estimation of actual intakes of individuals. - Design of diary can include prompts to ensure inclusion of different eating occasions (including snacks) and pictures to help gauge portion sizes therefore enabling more comprehensive data. - Reasonably cheap to collect data (although traditionally coding is time-consuming and expensive). - Use of standardised instructions and coding rules with skilled researchers will help improve accuracy. - Multiple food diaries - can be used to estimate usual intakes of individuals and distributions in a group if administered over a sufficient number of non-consecutive days and across seasons if relevant. - Misreporting can be minimised through researcher prompts or mid-diary contact (e.g. by telephone) to offer support, prompt, answer any queries and by standard checks/questions on collection for any missed entries. | - Not suitable for retrospective study, or distant past meals. - Labour-intensive for long term dietary intake or measuring intake of irregular consumed foods as would need multi-day diaries collected at multiple times spread over a year period. - Potential reactivity (likelihood of changes to usual food choice and omissions), increasingly likely as number of diary days increases. - Risk of lower completion rate of diaries as number of diary days increases. - Good literacy and numeracy needed unless research assistant or proxy (or carer/parent for children, elderly or translator) completes on behalf of participant. - High participant burden, especially if required to complete over several days and weigh foods. - Potential selection bias introduced as not all people are willing/able to complete a diet diary. - Moderate-to-high researcher burden, especially with manual coding which is very labour intensive requiring training and standardised quality assurance (QA)/quality control (QC) processes. - Generally expensive to code (solved to some extent by emerging technologies).   Estimated food diaries rely mostly on individual’s ability to describe portion sizes (photographs and food models can help address this). Participant may complete as a recall. |
| **24HR RECALL** |  |  |
| Retrospective, short-term method where details of foods and drinks consumed over previous 24 hours recalled. Can be administered by an interviewer (face to face or by telephone) following a standardised protocol. Can be administered as a single recall (for group-level assessment) or on multiple days (multiple recall) (required to capture individual variation). It can be used as a long term method if carried out over multiple phases. Can be completed online (see emerging technologies section). | - Provides detailed data leading to good estimates of short-term (past day) total dietary intake and total nutrient intake if completed well and if nutritional supplement use assessed. - Multiple 24hr recalls can be used to estimate usual intakes of individuals and distributions in a group if administered over a sufficient number of non-consecutive days and across seasons if relevant. - Allows collection of extra information (e.g. meal timing, frequency, location, brands eaten with whom, TV/computer/device etc.). - Provides flexible food and nutrient data that can be used in numerous types of analysis to answer a variety of research question. - Literacy and cultural issues minimised with trained interviewer. - If unannounced, element of surprise may lower reactivity i.e. changes to food intake because of measurement. - Moderate participant burden and high compliance depending on number of recall days. - Use of standardised protocols with skilled interviewer and multi-pass methods will help improve accuracy. | - Not suitable for measuring distant past meal or irregularly consumed foods. - Unsuitable for participants with memory issues. - Forgotten items are common. Intrusions (items not consumed) can also occur. - A single 24hr recall is unable to account for day to day variation. - Single 24 hr recall - fails to identify irregularly consumed foods and therefore intake of some nutrients can be underestimated. - Moderate-to-high researcher burden, especially with manual coding, requiring training and standardised QA/QC processes. - Expensive if face-to-face interview required and large number of participants. - 24 hour recalls rely completely on an individual’s ability to describe portion sizes (photographs and food models can help address this). |
| **FOOD FREQUENCY QUESTIONNAIRE (FFQ)** | | |
| Retrospective methods querying frequency over periods of time, questions relate to the frequency with which foods and drinks have been consumed over a long time period (weeks, months, and years). Can be ‘qualitative’ (frequency only), ‘semi-quantitative’ (estimated portion pre-assigned e.g. small, average, large) or ‘fully quantitative’ (portion size queried). Can be long (comprehensive, around 100 items queried or more) or short (also known as ‘screeners’ or a type of brief instrument). Can be interviewer- or self-administered, completed on paper (with potential scanning option) or online (see emerging technologies). | - Useful for estimating long term usual intakes of foods retrospectively; ranking participants into intake levels; estimating foods consumed irregularly. - Useful in large population studies as low researcher burden, potential low cost and low participant burden (higher response rates). - Long FFQs- potential for estimating usual dietary intake and total nutrient intake if portion size and dietary supplement use queried. - Short FFQs - suitable for estimating intake of small number of specific food items. Highly efficient approach when study has specific hypothesis to test, for example FFQ designed to determine calcium will contain only foods which provide calcium. - Short FFQs - low participant burden as quick to complete. - Coding generally less intensive. | - Not suitable for cross-cultural or cross-country comparisons unless comparable food lists included. - Short FFQs - not reliable for measuring total diet, total energy intakes and total nutrient intakes. - Requires good participant memory, literacy and numerical skills (e.g. to average intakes over long period of time) which can lead to inaccurate or subjective reporting. - Accurate reporting of frequencies and portions may be particularly difficult in children without parent/carer assistance. - Prone to mis-reporting if not carefully designed, especially for long FFQs. - Restricted to items that are specifically listed in the instrument. - It would not be possible to disaggregate foods which are listed together. - Requires specific algorithms in software to convert frequencies to nutrients. |
| **FOOD CHECKLISTS** | | |
| Also known as a type of ‘brief instrument’, ‘screeners’ or ‘short instruments’. Prospective, short-term method where specified foods and drinks are ticked from a list as they are consumed over a day or number of days; frequency can be queried; option to query portion sizes or pre-assign them. This is less used method that has a lot of strengths and weaknesses in common with the longer FFQ. Can be completed online (see emerging technologies). | - Can be suitable for estimating intakes of specific foods or nutrients occurring in high levels in specific foods. - Can be useful for comparing short-term group mean intakes of a small group of items, patterns, change over time, compliance with dietary guidelines and providing dietary advice. - Low researcher and participant burden, low cost. - Coding generally simple. | - Generally brief so unsuitable for measuring total diet, total nutrient and energy intake. - Not suitable for cross-cultural or cross-country comparisons unless comparable food lists included. - Restricted to items that are specifically listed in the instrument. |
| **DIET HISTORIES** |  |  |
| Combination of short-term and long-term methods, usually 24hr recall, FFQ and food diary; more often used in a clinical setting by experienced dieticians to provide in-depth assessment at individual level. | - Potential for estimating usual intake of foods and nutrients if over long period (e.g. 1 month or more) depending on balance of days. - Ability to assess meal patterns and food preparation over extended period of time. - Some diet history instruments have been automated and adapted for self-administration. | - Protocols tend to vary as no agreed standardised approach available, food records and 24HRs are sometimes used to [check](http://dietassessmentprimer.cancer.gov/glossary.html#calibration) the diet history with detailed questions about usual eating patterns by meal. - The meal-based approach is not suitable for individuals who have no regular eating pattern. - High participant burden. - High researcher burden, as the interview and the food list component each can take up to an hour to complete. - Complex analysis processes required. - Generally expensive as it requires trained interviewer and coding the data. |
| **EMERGING TECHNOLOGIES (Based on traditional methods)** | | |
| Dietary data collected and processed making use of hardware plus software (e.g. devices such as sensors and optical readers) or software such as web-based versions and apps based on traditional DATs. More advanced method of collecting data, which could be based on traditional methods (FFQs, food diary or 24 h recall). It is a rapidly evolving area. | - Potential for providing ‘real time’ food/nutrient results output. - Potential for enhanced portion size and food waste estimation (using digital capturing of meals and photos). - Potential for low to moderate participant burden and higher participant motivation (depending on the participants’ technological ability and the technology itself). - Potential to prompt recording to reduce mis-recording. - Online versions of recalls, diaries and FFQs may be useful for large sample sizes due to lower researcher burden (e.g. interviewers, data entry, manual coding, printing may not be required). | - As with all recently developed DATs, validation/calibration data may not yet have been generated for evaluating DAT quality. - Likely to inherit similar measurement error to non-adapted version (e.g. paper-based vs web-based FFQ). - Internet, computer and mobile-technology skills and access required. - Participant training required if tool not intuitive. - Potential high initial cost of specialised equipment and software programming. |
